# Synthesis and Immunological Evaluation of a Single Molecular Construct MUC1 Vaccine Containing l-Rhamnose Repeating Units

**DOI:** 10.3390/molecules25143137

**Published:** 2020-07-09

**Authors:** Md Kamal Hossain, Abhishek Vartak, Steven J. Sucheck, Katherine A. Wall

**Affiliations:** 1 Department of Medicinal and Biological Chemistry, University of Toledo, Toledo, OH 43606, USA; khossain@umich.edu; 2 Department of Chemistry & Biochemistry, University of Toledo, Toledo, OH 43606, USA; vartaka@mskcc.org (A.V.); steve.sucheck@utoledo.edu (S.J.S.)

**Keywords:** cancer immunotherapy, cancer vaccine, anti-rhamnose antibodies, rhamnose, liposomes, MUC1

## Abstract

A rhamnose targeting strategy for generating effective anticancer vaccines was successful in our previous studies. We showed that by utilizing natural anti-rhamnose antibodies, a rhamnose-containing vaccine can be targeted to antigen-presenting cells, such as dendritic cells. In this case, rhamnose (Rha) was linked directly to the liposomes bearing the antigen. However, in the current approach, we conjugated a multivalent Tri-Rha ligand with the antigen itself, making it a single component vaccine construct, unlike the previous two-component vaccine construct where Rha cholesterol and Mucin1 (MUC1) antigen were both linked separately to the liposomes. Synthesis required the development of a linker for coupling of the Rha-Ser residues. We compared those two systems in a mouse model and found increased production of anti-MUC1 antibodies and more primed antigen-specific CD4+ T cells in both of the targeted approaches when compared to the control group, suggesting that this one-component vaccine construct could be a potential design used in our MUC1 targeting mechanisms.

## 1. Introduction

Mucin1 (MUC1) is a cell-surface-associated transmembrane protein, containing multiple, twenty amino acid long, variable number tandem repeats (VNTRs) [1,2]. Generally, it is hyper-glycosylated in normal cells but hypo-glycosylated in cancerous cells [3,4,5]. This structural difference makes it an attractive target for anticancer vaccine preparation. Different laboratories have used different approaches to increase the immunogenicity of MUC1 [6,7,8,9,10,11,12,13]. We utilized a MUC1-Tn antigen to conjugate with an adjuvant, such as toll-like receptor ligands (e.g., TLR-2 ligand, Pam_3_Cys, or Pam_3_CSK_4_), for making our vaccine preparation. The vaccine conjugate was then incorporated into liposomes before immunizing mice [14,15].

Once dendritic cells (DCs) encounter the vaccine construct, they become mature dendritic cells that can then internalize the vaccine, and process and present antigen through their major histocompatibility complex (MHC) [16,17]. MHC-II molecules can present peptide antigen to CD4+ T cells and MHC-I molecules can present short peptides (usually 8–11 amino acids long) to CD8+ T cells [18]. Antibodies that recognize the vaccine can bind to Fc receptors on DCs and enhance maturation and uptake. We showed in 2010 that the immunogenicity of a Tn tumor antigen-based cancer vaccine could be increased with the conjugation of l-rhamnose (Rha) as a targeting ligand [19]. Mice were immunized with Rha-ovalbumin or Rha-Ficoll to generate anti-Rha antibodies prior to vaccination. We then incorporated different MUC1 B cell and T cell peptide epitopes, bound to Pam_3_Cys, into liposomes that had l-Rha-cholesterol, bound on the same liposomal surface, and showed that vaccine effectiveness could be enhanced in the presence of anti-Rha antibodies [12,14]. This approach caused production of more MUC1 specific antibody, that can bind to the MUC1 expressing tumor cells [12], in order to destroy them through antibody-dependent cell-mediated cytotoxicity (ADCC). The generation of anti-MUC1 CD8+ T cells was also enhanced.

Rhamnose is a bacterial sugar that often generates natural anti-rhamnose antibodies (anti-Rha) in humans. It has been found that anti-Rha antibodies are one of the most predominant natural antibodies present in human serum [20,21]. In our recent studies, we utilized passive transfer of natural human anti-Rha antibodies into mice to enhance the MUC1-based vaccine’s immunogenicity [15]. We demonstrated increased Rha-antigen uptake, and presentation by DCs, in vitro in the presence of natural human anti-rhamnose antibodies [15]. Enhancement seems to involve Fc receptors, since the enhancement depends on the isotype distribution of the anti-rhamnose antibodies present [12,15]. We used a two-component vaccine preparation, where rhamnose cholesterol, and Pam_3_CSK_4_-MUC1 Tn vaccine constructs, were both incorporated into liposomes [15,22]. We hypothesized that a single molecule construct, containing Pam_3_CSK_4_ as an adjuvant, and MUC1 antigen, directly conjugated to rhamnose as a targeting ligand, would lead to precise targeting, as well as enhanced immune response. In this study, we compared these two approaches; the multi-epitope single-component liposomal system, and the earlier two-component system.

## 2. Results and Discussion

For the new vaccine construct, we envisioned that a multivalent rhamnose construct would be better than a single rhamnose direct construct, as had previously been used [19]. We attempted the synthesis of a peptide construct, consisting of three copies of Rha-Ser attached to the *C*-terminal end of a MUC1-VNTR sequence during solid phase peptide synthesis (SPPS), followed by the attachment of 6-azido-hexanoic acid to the *N*-terminal end. Unfortunately, multiple attempts at synthesizing the peptide were unsuccessful. We speculated that the loading of three consecutive, sterically bulky, Rha-Ser amino acid residues on the SPPS resin was the primary cause of the synthesis failure. A new synthetic strategy was adopted, with a Fmoc-NH-PEG_2_-CH_2_COOH linker at the *C*-terminal end [23], followed by three copies of Rha-Ser, a MUC1-VNTR sequence, and 6-azido-hexanoic acid at the *N*-terminal end. The azide on the *N*-terminus allowed for coupling to Pam_3_CSK_4_-DBCO and incorporation into liposomes. For comparison, a liposomal vaccine containing Pam_3_CSK_4_-MUC1 VNTR was targeted with Rha-Cholesterol. Neither construct contained a Tn modification on the VNTR.

**Scheme 1:**
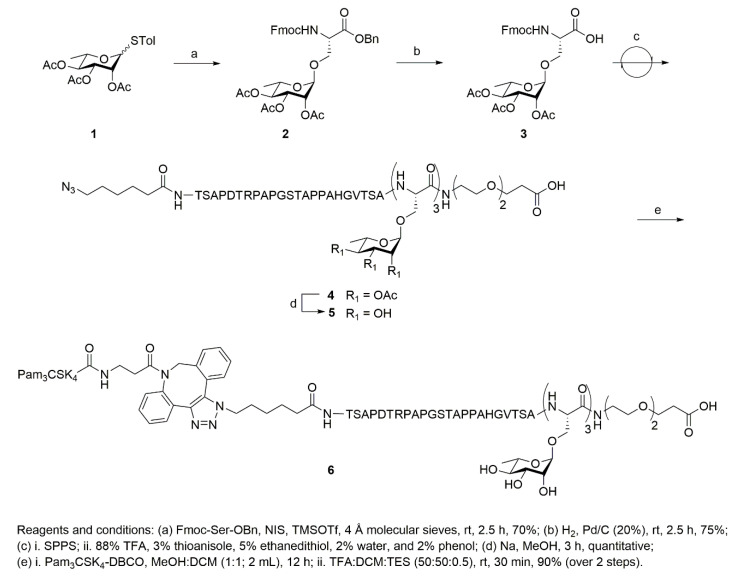
Synthesis of single molecule construct of Pam_3_CSK_4_-DBCO-MUC1-(Rha)_3_ sequence.

Pam_3_CSK_4_-DBCO was synthesized as previously described [15]. The targeting ligand, l-rhamnose, was coupled with Fmoc-Ser-OBn to incorporate the corresponding modified amino acid into the solid phase peptide synthesis (SPPS) (Scheme 1). Fmoc-Ser-OBn was glycosylated with *S*-4-Methylphenyl-2,3,4-tri-*O*-acetyl 1-thio-α-d-rhamnoside **1** using the NIS-TMSOTf promoter system to afford compound **2** in 70% yield (Scheme 1, Figures S1–S3) [24,25]. The benzyl group was removed using Pd-catalyzed hydrogenation to afford modified amino acid building blocks (**3**) for SPPS (Figures S4–S6) [26]. 

**Scheme 2:**
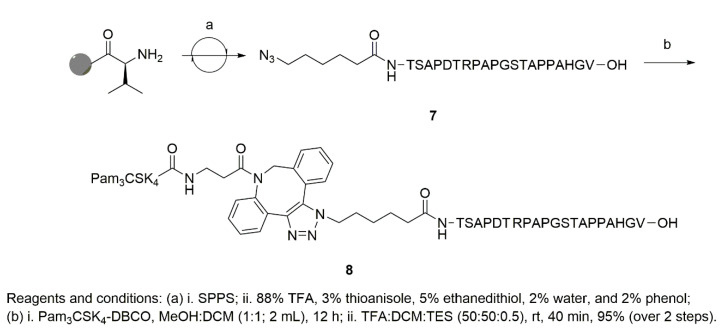
Synthesis of Pam_3_ CSK_4_-DBCO-MUC1 sequence.

Azide-terminated peptides (**4** and **7**, Scheme 1 and Scheme 2) were synthesized using a CEM Liberty Blue automated microwave peptide synthesizer. The synthesis of **4** was achieved with a PEG linker at the *C*-terminal end, followed by three repeating units of **3**, a MUC1-VNTR sequence, and 6-azido-hexanoic acid. The loading of three consecutive units of **3** on SPPS was extremely challenging and required multiple cycles for complete coupling. To synthesize the cycloaddition product, Pam_3_CSK_4_-DBCO was dissolved in MeOH:DCM (1:1, 2 mL) along with azide-terminated peptide (**4**, **7**) and stirred overnight, under N_2_, at room temperature (RT). The reaction mixture was evaporated, and global deprotection was achieved using a cleavage cocktail of DCM:TFA:TES to produce the final conjugates, **6** at 90% (Figure S7), and **8** at 95% (Figure S8), respectively. 

The liposomes contained 80% DPPC and 20% cholesterol and were made as described in [12,27]. The vaccine for comparison, referred to as the single rhamnose vaccine, contained the same components, except that the three rhamnose-serines were omitted from the MUC1 antigen. The liposomes contained 80% DPPC, 10% cholesterol, and 10% Rha-TEG-Cholesterol [12].

Four groups of four mice were used for immunization purposes. Two groups of mice received anti-Rha antibody prior to immunization with either the tri-Rha vaccine or the single rhamnose vaccine. The anti-Rha antibody was affinity purified, as described in [15], from pooled human serum. Another group of mice received pass-through antibody, which contains all other human antibodies (except anti-Rha antibodies), before receiving the tri-Rha vaccine. The fourth group of mice received neither the antibody nor the vaccine.

Group A: Anti-Rha antibody (1 h earlier) + Pam_3_CSK_4_ -DBCO-MUC1-(Rha)_3_ conjugate **6** (10 nmoles per mouse)**,** cholesterol (20%), and DPPC (80%).

Group B: Pass-through antibody (1 h earlier) + Pam_3_CSK_4_ -DBCO-MUC1-(Rha)_3_ conjugate **6** (10 nmoles per mouse), cholesterol (20%), and DPPC (80%).

Group C: Anti-Rha antibody (1 h earlier) + Pam_3_CSK_4_ -DBCO-MUC1 conjugate **8** (10 nmoles per mouse), Rha-cholesterol (10%), cholesterol (10%), and DPPC (80%).

Group D: Non-immunized mice.

**Figure 1:**
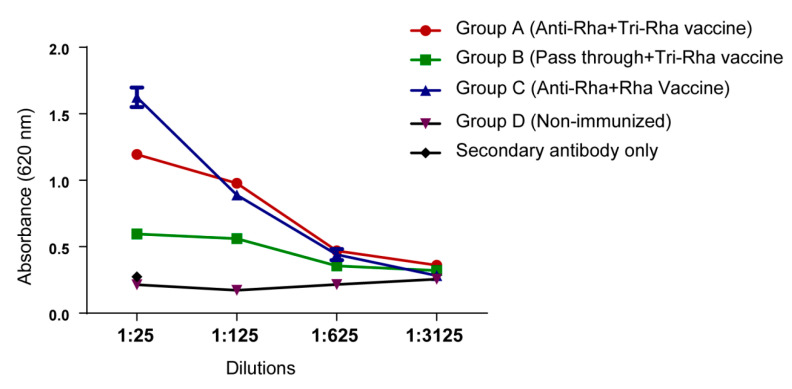
Anti-Mucin1 (MUC1) antibody production through ELISA. Anti-MUC1 antibodies in pooled serum were assayed by ELISA on MUC1-coated plates. Secondary antibody was anti-mouse IgG (H + L).

The mice were boosted with the same vaccine constructs every two weeks and were bled seven days after the second boost to measure the presence of anti-MUC1 antibody (Figure 1). Sera collected from group A and group C mice showed much higher MUC1 antibody production compared with the mice receiving pass-through antibody and the other non-immunized mice.

**Figure 2:**
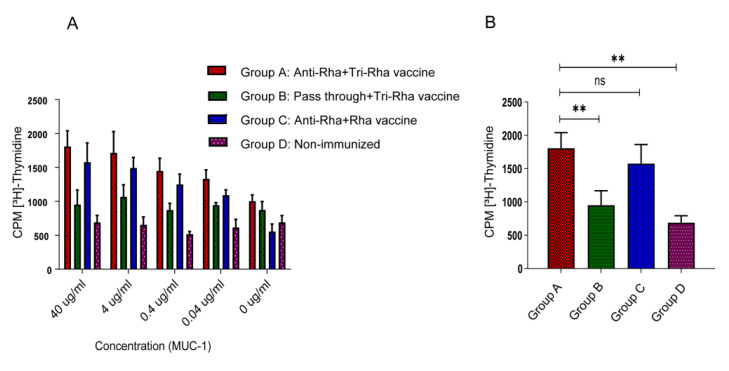
CD4+ T cell proliferation assay. (**A**) MUC1 concentration-dependent CD4+ T cell proliferation. (**B**) CD4+ T cell proliferation in the presence of 40 µg/mL of MUC1 antigen. Statistical significance was assessed using one-way analysis of variance (ANOVA). Analysis was performed with Graph Pad prism software version 8.4.2 (** *p* < 0.001, ns > 0.05).

The mice were sacrificed seven days after the second boost and purified spleen CD4+ T cells were collected to measure the cell proliferation present in bone-marrow-derived dendritic cells, and the different concentrations of MUC1 antigen (Figure 2). Higher CD4+ T cell proliferation was observed in the group A and group C mice that had received anti-Rha antibodies prior to vaccination, compared with the mice that had received pass-through antibodies (group B).

Both the tri-rhamnose vaccine and the single rhamnose-cholesterol vaccine gave similar enhancements in antibody production and MUC1-specific T cell stimulation. The amounts of Rha on each vaccine construct differed, with a 3:1 ratio of Rha to antigen in the directly conjugated vaccine, and a 30:1 ratio of Rha to antigen in the Rha-cholesterol vaccine. However, in both cases, multiple copies of Rha are accessible to anti-Rha antibodies on each liposome. Evidently, once sufficient anti-Rha antibody has bound to the liposome, additional binding sites do not confer an advantage. Both vaccines tested here gave similar levels of specific antibody to other Pam_3_CSK_4_-containing vaccines [28]; however, we avoided the addition of a strong adjuvant such as complete Freund’s adjuvant.

The synthesis of three units of Rha-Ser-containing peptide on SPPS was challenging as a consequence of steric bulk and required a PEG linker, at the *C*-terminal end of the peptide, to facilitate the synthesis. This adds to the expected expense of producing a vaccine with this design. It was approximately 10 times more expensive to prepare when compared with the rhamnose-cholesterol vaccine.

## 3. Materials and Methods

### 3.1. Mouse Strain and Immunization

Six to eight-week-old female C57BL/6 mice were obtained from Jackson Laboratories, Bar Harbor, Maine. Groups of 4 mice were each injected with 10 µg of either affinity-purified anti-Rha antibodies or pass-through antibodies containing all other human antibodies except anti-Rha antibodies in 100 µL of PBS. Pooled human serum (ZenBio Inc., Research Triangle Park, NC, USA) was purified as described in [15]. After 1 h, the mice were then immunized with 10 nmoles of antigen in either the Tri-Rha vaccine or the single Rha-vaccine in liposomal formulations (100 µL in PBS) intraperitoneally. The mice were boosted every two weeks, following the same procedure, with newly formulated liposomes. Animals were kept at the animal facility of the University of Toledo in specific, pathogen-free, housing following National Institutes of Health guidelines with oversight by the University of Toledo Institutional Animal Care and Use Committee, protocol 106051, latest approval 04/12/2017.

### 3.2. ELISA Assay

The mice were bled by submandibular lancet 7 days after each immunization, and the serum was separated and pooled within each group. A 96-well Immulon 4HBX plate was coated with 2 µg/mL of MUC1 antigen in PBS and incubated overnight at 4 °C. The next day, the plate was washed five times with washing buffer (PBS with 0.1% Tween-20) and blocking buffer was added at a concentration of 15 µg/mL of BSA in PBS for 2 h. After washing five more times, different dilutions of mice sera were added and incubated for an hour at room temperature. After washing again, HRP-conjugated goat anti-mouse IgG (H + L) secondary antibody (1:10,000, Invitrogen) was added and incubated at room temperature. Finally, the plate was washed again before adding TMB substrate (BioFX, Inc., Eden Prairie, MN, USA) and the absorbance at 620 nm was measured.

### 3.3. Proliferation Assay

The mice were euthanized for spleen cell harvest 7 days after the second boost. A T cell proliferation assay was performed according to the protocol described earlier [15]. Briefly, CD4+ T cells were positively isolated using a Dynabeads Flowcomp Mouse CD4 kit (Invitrogen). The cells were suspended in T cell medium, which consisted of RPMI 1640 with l-glutamine, 10% heat-inactivated fetal bovine serum, 5 × 10^−5^ M β-mercaptoethanol, 2 mM l-glutamine, 20 mM HEPES at pH 7.4, 100 U/mL penicillin, 100 µg/mL streptomycin, and 1% media additions (0.06 g of folic acid, 0.36 g of l-asparagine, 1.16 g of l-arginine, 2.16 g of l-glutamine, and 1.10 g of sodium pyruvate in 100 mL of PBS). Bone-marrow-derived dendritic cells (BMDCs) were prepared according to the procedure of Matheu et al. [29]. In a 96-well plate, different concentrations of MUC1 peptide were mixed with 2 × 10^4^ BMDCs and incubated for 30 min. Then, 2 × 10^5^ CD4+ cells, pooled within each group, were added to give a final volume of 200 µL. The cells were incubated for 72 h at 37 °C and 5% CO_2_. [^3^H]-thymidine (Moravek, Inc., Brea, CA, USA) was then added at 40 μCi/mL. After overnight incubation, cells were harvested onto a glass fiber filter plate, scintillation fluid was added, and [^3^H]-thymidine incorporation was determined on a TopCount scintillation counter.

### 3.4. Synthesis of Pam_3_CSK_4_-DBCO-MUC1

All fine chemicals and solvents were obtained from Fisher Scientific and Sigma-Aldrich. The solvents for reactions were purified using a PureSolv MD5 Solvent Purification System (SPS). Reactions were monitored using thin-layer chromatography (silica gel 60, f254) and spots were observed by UV light or by charring (5% H_2_SO_4_ in MeOH). Preloaded Wang resins and Fmoc amino acids were obtained from AAPPTEC, AnaSpec, or Chem-Impex International, Inc. Flash column chromatography was performed on silica gel (230–400 mesh) obtained from Sorbent Technologies using solvents as received. ^1^H-NMR were carried out using an Avance III 600 MHz spectrometer (Bruker Corporation, Billerica, MA, USA)using residual CHCl_3_ or MeOD as internal references. ^13^C NMR were recorded at 150 MHz using residual CHCl_3_ or MeOD as internal references. Matrix-Assisted Laser Desorption/Ionization (MALDI) was performed on an ultrafleXtreme instrument (Bruker Corporation, Billerica, MA, USA). DHB (2,5-Dihydroxybenzoic acid) was used as a matrix for MALDI analysis. High resolution mass spectroscopy (HRMS) was performed on a Micromass Q-TOF 2 instrument.

#### 3.4.1. Synthesis of *N*-[(9*H*-fluoren-9-yl)methoxycarbonyl]-*O*-((2,3,4-tri-O-acetyl)-α-l-rhamnopyranosyl)-l-serine Phenylmethyl Ester (Fmoc-l-Ser(Ac_3_-α-Rhamose)-OBn) (**2**)

Compound **1** (1.5 g, 3.8 mmol) and Fmoc-Ser-OBn (1.44 g, 3.44 mmol) were dissolved in dry DCM (22 mL) and stirred with 4 Å molecular sieves (150 mg) under N_2_ at room temperature. NIS (1.2 g, 5.1 mmol) and TMSOTf (210 µL, catalytic) were added to the reaction after 30 min. The reaction was monitored by TLC and appeared complete after 2.5 h. The reaction was diluted with DCM (25 mL) and filtered through Celite 545. The filtrate was washed with aq. NaHCO_3_ (20 mL, ×2), brine solution (10 mL, ×2), and water (10 mL, ×2). The organic layer was separated, dried over anhydrous sodium sulfate, and subjected to silica gel column chromatography (Hex:EtOAc, 8:2) to afford the final product as a white solid **2** (1.8 g, 70%).

^1^H-NMR (600 MHz, CDCl_3_): δ 1.12 (d, *J* = 6.18 Hz, 3H, Rha-CH_3_), 2–2.17 (s, 9H, -OAc H), 3.71 (m, 2H, Ser -CH_2_), 4.20 (dd, *J* = 9.9, 2.94 Hz, 1H), 4.26 (t, *J* = 7.26 Hz, 1H, Ser-CH), 4.40 (m, 2H), 4.67 (d, *J* = 8.64 Hz, 1H), 4.75 (s, 1H), 5.05 (t, *J* = 9.9 Hz, 1H), 5.17 (dd, *J* = 10.08, 3.42 Hz, 1H), 5.28(s, 2H, -Benzyl CH_2_), 5.77 (d, *J* = 8.64, 1H, H-1), 7.3–7.8 (m, 13H). ^13^C-NMR (150 MHz, CDCl_3_): δ 17.48, 21.03, 21.11, 47.27, 54.34, 66.99, 67.71, 67.95, 98.2, 69.12, 69.62, 70.94, 97.85, 120.18, 125.46, 127.33, 127.393, 128.5, 128.78, 128.91, 134.07, 135.11, 141.49, 143.95, 156.20, 169.71, 170.12, 170.31. ESI-MS [M + Na] *m*/*z*: calcd for C_37_H_39_NNaO_12_, 712.70; found, 712.50.

#### 3.4.2. Synthesis of *N*-[(9*H*-fluoren-9-yl)methoxycarbonyl]-*O*-((2,3,4-tri-*O*-acetyl)-α-l-rhamnopyranosyl)-l-serine (Fmoc-l-Ser(Ac_3_-α-Rhamose)-OH) (**3**)

Pd/C (20%) (0.5 g) was added to a solution of **2** (1.6 g, 2.3 mmol) in anhydrous MeOH (20 mL). The reaction mixture was stirred under H_2_ at room temperature. The Fmoc group on the primary amine started to fall off after 2.5 h along with the desired benzyl deprotection. The reaction was diluted with methanol (20 mL) and filtered through Celite 545. The filtrate was subjected to silica gel column chromatography (CHCl_3_:EtOH) to obtain the desired product as a white foamy solid **3** (0.78 g, 75%). Four hundred milligrams of starting material, **2**, was recovered.

^1^H-NMR (600 MHz, CDCl_3_): δ 1.22 (d, *J* = 6.18 Hz, 3H, Rha-CH_3_), 1.98–2.16 (s, 9H, -OAc H), 3.74 (m, 1H, Ser -CH_2_), 3.85 (m, 1H, Ser -CH_2_), 4.25 (m, 2H, Ser -CH), 4.41 (t, *J* = 6.24 Hz, 1H), 4.68 (d, *J* = 8.34 Hz, 1H), 4.78 (s, 1H), 5.05 (t, *J* = 9.96 Hz, 1H), 5.22 (dd, *J* = 10.08, 3.18 Hz, 1H), 5.31 (m, 1H), 5.96 (d, *J* = 8.4 Hz, 1H, H-1), 7.26–7.78 (aromatic 8H, Fmoc-H). ^13^C-NMR (150 MHz, CDCl_3_): δ 17.5, 20.96, 21.13, 47.28, 54.02, 67.04, 67.73, 69.36, 69.73, 71.04, 97.79, 120.21, 125.43, 127.36, 127.96, 141.5, 143.92, 156.42, 170.27, 170.47, 170.62, 172.21. HRMS [M + Na] *m*/*z*: calcd for C_30_H_33_NNaO_12_, 622.1900; found, 622.1903.

#### 3.4.3. Synthesis of Azide-Terminated Peptide Sequences, (**4**) and (**7**)

The peptides were synthesized on a CEM Liberty Blue Automated Microwave Peptide Synthesizer, using the Fmoc strategy. The peptide synthesis was performed on a 100 μmol scale with the help of *N*,*N*′-diisopropylcarbodiimide (DIC) and 1-hydroxybenzotriazole (HOBt) as coupling agents, and 25% piperidine in dimethylformamide (DMF) for *N_α_*-Fmoc group deprotection. The Fmoc-NH-PEG_2_-CH_2_COOH was loaded on the Wang resin followed by three repeating units of Fmoc-l-Ser(Ac_3_-α-Rhamose)-OH **3**, and the Fmoc-protected amino acid residues for the synthesis of peptide **4** as indicated in Scheme 1. For the final coupling, 6-azido-hexanoic acid was used on the solid phase synthesizer. A similar protocol was followed to obtain peptide **7**, starting from pre-loaded Fmoc-Val-Wang resin (Scheme 2).

Following the completion of the synthesis, both cleavage and side-chain deprotection reactions were manually performed using a modified reagent K cocktail (88% TFA, 3% thioanisole, 5% ethanedithiol, 2% water, and 2% phenol). The peptides were then precipitated out and washed using ice-cold anhydrous *tert*-butyl ether. The precipitated and washed peptides were then dissolved in 25% acetonitrile in water for analysis and purification. Prior to preparative-scale HPLC purification, analytical HPLC (Beckman System Gold) was performed using a Vydac C18 column to identify the peptide. The purity of the crude preparation was checked by gradient elution (5–60% acetonitrile) with a single peak at 214 nm (solvent A = 0.085% TFA in water; solvent B = 98% acetonitrile and 0.085% TFA in water). The analysis was performed using a MALDI-TOF/TOF mass spectrometer (Model No. 4800; AB SCIEX, Framingham, MA). Following HPLC purification (Waters Corporation), using a Vydac preparative C18 column, the purity (>95%) was further assessed by analytical HPLC and mass spectrometry, as performed above. The peptides were lyophilized and stored at −20 °C. Peptide **4**: MALDI-TOF: [M + H] *m*/*z* calcd for C_147_H_227_N_35_O_64_, 3507.56; found 3508.64. Peptide **7**: MALDI-TOF: [M + H] *m*/*z* calcd for C_86_H_136_N_28_O_29_, 2026.1793; found 2026.3013.

#### 3.4.4. Synthesis of the Single Molecule Construct of Pam_3_CSK_4_-DBCO-MUC1-(Rha)_3_ (**6**)

Compound **5** (1.2 mg, 0.4 µmol) and Pam_3_CSK_4_-DBCO (1.1 mg, 0.5 µmol) were dissolved in anhydrous DCM:MeOH (1:1, 2 mL), and stirred overnight under N_2_ at room temperature. The solution was evaporated and the cycloaddition product (1.5 µmol) was dissolved in a cleavage cocktail of DCM:TFA:TES (50:50:0.5, 1 mL). The reaction was stirred for 40 min at room temperature in an N_2_ atmosphere. The solvent was evaporated, and the remaining solution was added to cold ether (−10 °C, 5 mL). The solution was kept at −20 °C overnight for precipitation of the target compound. The precipitate was centrifuged down, followed by two washings with cold ether and then dried under a high vacuum to obtain compound **6** (2 mg, 90%). MALDI-TOF: [M + H] *m*/*z* calcd for C_228_H_381_N_47_O_68_S, 4900.757; found 4900.659.

#### 3.4.5. Synthesis of Pam_3_CSK_4_-DBCO-MUC1 (**8**)

Compound **7** (2.0 mg, 0.9 µmol) and Pam_3_CSK_4_-DBCO (2.4 mg, 1.0 µmol) were subjected to a similar procedure of cycloaddition and deprotection, as mentioned above, to obtain compound **8** (3.2 mg, 95%). MALDI-TOF: [M + H] *m*/*z* calcd for C_185_H_306_N_40_O_42_S, 3794.622; found 3794.938.

## 4. Conclusions

In conclusion, a single-component peptide construct containing lipopeptide adjuvant Pam_3_CSK_4_, a MUC1-VNTR sequence, and three consecutive units of the antibody-recruiting molecule Rha-Ser was successfully synthesized and formulated into a liposomal delivery system. The synthesis required the synthesis of an Fmoc-protected Rha-Ser building block that could be prepared on a multi-gram scale. In addition, the development of a PEG linker at the *C*-terminal end of the peptide was critical in order to accomplish the synthesis of the *C*-terminal repeat of the Rha-Ser. While we were pleased to obtain the desired materials for study, it should be noted that the peptide synthesis was lower yielding in comparison with the previously reported MUC1 azido-peptides. In the current approach, the peptide synthesis was conducted on the rather large 100 micromole scale and yielded only about 2.5 mg of purified peptide even though triple couplings of amino acids were used. In retrospect, the yield of the peptide synthesis could potentially be improved if the difficult-to-couple Rha-Ser residues were introduced at the end of the synthesis. In addition, the inclusion of less sterically hindered intervening peptides may also have been beneficial.

Vaccination studies with a single construct liposomal vaccine have shown comparable antigen specific antibody production and antigen-primed CD4+ T cell proliferation to that of a two-component, Rha-bearing, liposomal vaccine preparation. The single component vaccine is therefore an alternative approach to MUC1-based vaccine preparation, but one that may need modification to achieve cost effectiveness.

## Supplementary Materials

The following are available online, Figure S1: ESI-MS data on *N*-[(9H-fluoren-9-yl)methoxycarbonyl]-*O*-((2,3,4-tri-*O*-acetyl)-α-l-rhamnopyranosyl)-l-serine phenylmethyl ester **2**; Figure S2: ^1^H-NMR of *N*-[(9H-fluoren-9-yl)methoxycarbonyl]-*O*-((2,3,4-tri-*O*-acetyl)-α-l-rhamnopyranosyl)-l-serine phenylmethyl ester **2**; Figure S3: ^13^C-NMR of *N*-[(9H-fluoren-9-yl)methoxycarbonyl]-*O*-((2,3,4-tri-*O*-acetyl)-α-l-rhamnopyranosyl)-l-serine phenylmethyl ester **2**; Figure S4: HRMS data on *N*-[(9H-fluoren-9-yl)methoxycarbonyl]-*O*-((2,3,4-tri-*O*-acetyl)-α-l-rhamnopyranosyl)-l-serine **3**; Figure S5: ^1^H-NMR of *N*-[(9H-fluoren-9-yl)methoxycarbonyl]-*O*-((2,3,4-tri-*O*-acetyl)-α-l-rhamnopyranosyl)-l-serine **3**; Figure S6: ^13^C-NMR of *N*-[(9H-fluoren-9-yl)methoxycarbonyl]-*O*-((2,3,4-tri-*O*-acetyl)-α-l-rhamnopyranosyl)-l-serine **3**; Figure S7: MALDI-TOF data on the single molecule construct of Pam_3_CSK_4_-DBCO-MUC1 -(Rha)_3_ sequence **6**; Figure S8: MALDI-TOF data on the single molecule construct of Pam_3_CSK_4_-DBCO-MUC1 **8**.
