# Supplementary material for: Synthesis and Immunological Evaluation of a Single Molecular Construct MUC1 Vaccine Containing l-Rhamnose Repeating Units
Source: Molecules. 2020 Jul 9;25(14):3137. doi: 10.3390/molecules25143137 (PMC7397004; doi:10.3390/molecules25143137)

Supplementary information for:

Synthesis and immunological evaluation of a single molecular construct MUC1 vaccine containing L-rhamnose repeating units

Md Kamal Hossain, Abhishek Vartak, Steven J. Sucheck and Katherine A. Wall

Figure S1. ESI-MS data of *N*-[(9H-fluoren-9-yl)methoxycarbonyl]-*O*-((2,3,4-tri-*O*-acetyl)-α-L-rhamnopyranosyl)-L-serine phenylmethyl ester **2**

Figure S2. ^{1}H NMR of *N*-[(9H-fluoren-9-yl)methoxycarbonyl]-*O*-((2,3,4-tri-*O*-acetyl)-α-L-rhamnopyranosyl)-L-serine phenylmethyl ester **2**

Figure S3. ^{13}C NMR of *N*-[(9H-fluoren-9-yl)methoxycarbonyl]-*O*-((2,3,4-tri-*O*-acetyl)-α-L-rhamnopyranosyl)-L-serine phenylmethyl ester **2**

Figure S4. HRMS data of *N*-[(9H-fluoren-9-yl)methoxycarbonyl]-*O*-((2,3,4-tri-*O*-acetyl)-α-L-rhamnopyranosyl)-L-serine **3**

Figure S5. ^{1}H NMR of *N*-[(9H-fluoren-9-yl)methoxycarbonyl]-*O*-((2,3,4-tri-*O*-acetyl)-α-L-rhamnopyranosyl)-L-serine **3**

Figure S6. ^{13}C NMR of *N*-[(9H-fluoren-9-yl)methoxycarbonyl]-*O*-((2,3,4-tri-*O*-acetyl)-α-L-rhamnopyranosyl)-L-serine **3**

Figure S7. MALDI-TOF data of single molecule construct of Pam_3CSK_4-DBCO-MUC1 -$(Rha)_3$ sequence **6**

Figure S8. MALDI-TOF data of single molecule construct of Pam_3CSK_4-DBCO-MUC1 **8**

Figure S1. ESI-MS data of *N*-[(9H-fluoren-9-yl)methoxycarbonyl]-*O*-((2,3,4-tri-*O*-acetyl)-α-L-rhamnopyranosyl)-L-serine phenylmethyl ester (**2**)

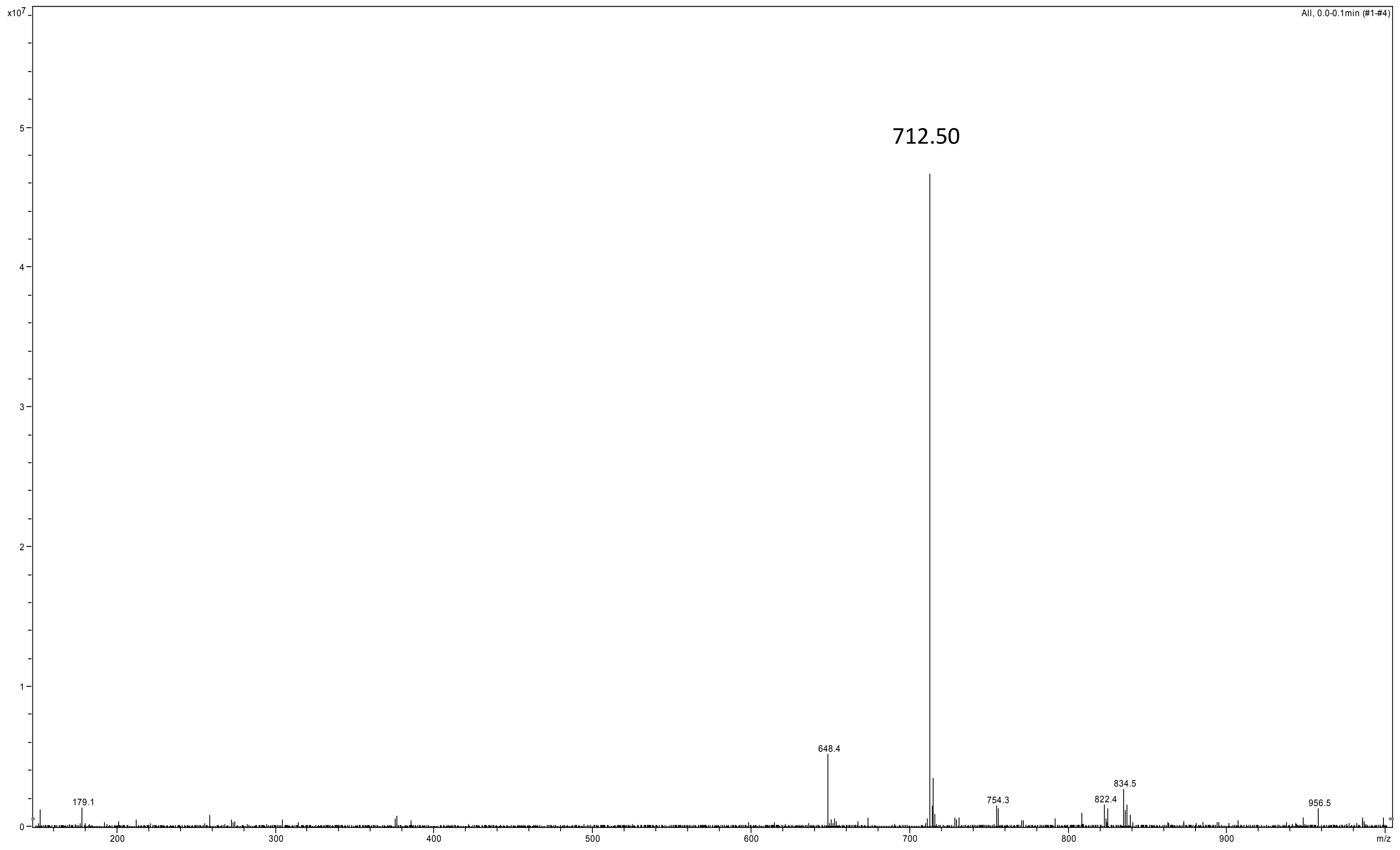

Figure S2. ^{1}H NMR of *N*-[(9H-fluoren-9-yl)methoxycarbonyl]-*O*-((2,3,4-tri-*O*-acetyl)-α-L-rhamnopyranosyl)-L-serine phenylmethyl ester (**2**)

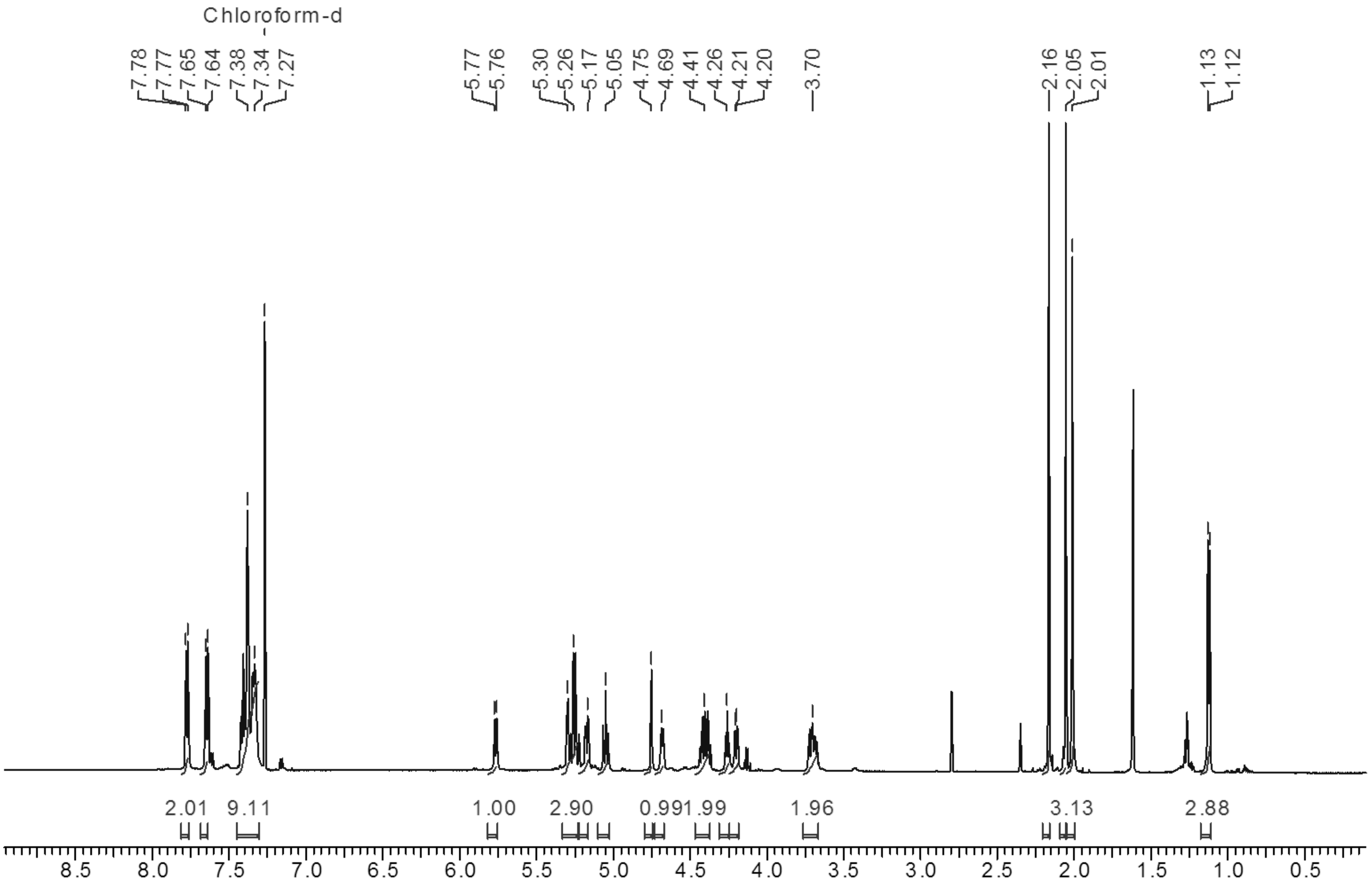

Figure S3. ^{13}C NMR of *N*-[(9H-fluoren-9-yl)methoxycarbonyl]-*O*-((2,3,4-tri-*O*-acetyl)-α-L-rhamnopyranosyl)-L-serine phenylmethyl ester (**2**)

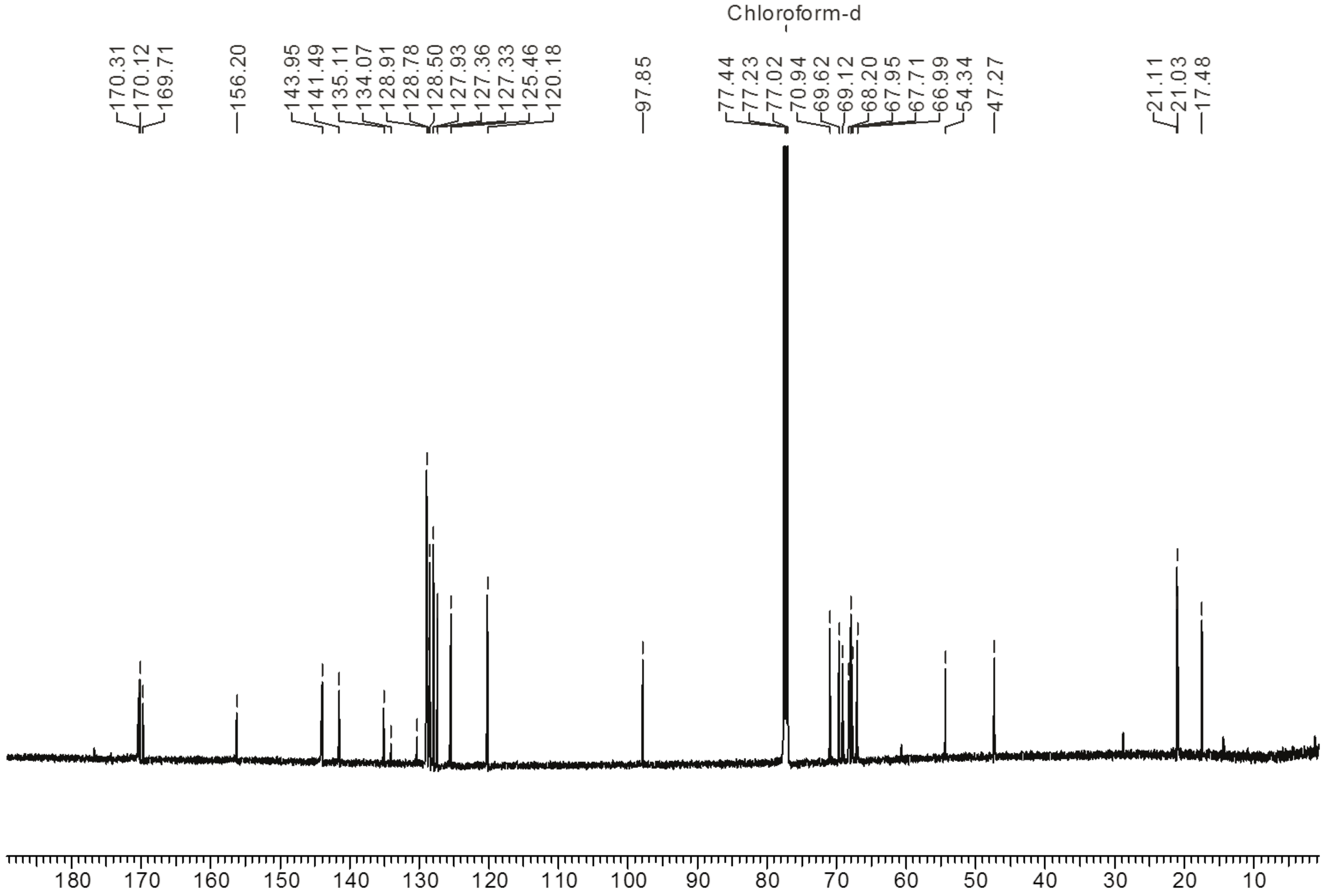

Figure S4. HRMS data of *N*-[(9H-fluoren-9-yl)methoxycarbonyl]-*O*-((2,3,4-tri-*O*-acetyl)-α-L-rhamnopyranosyl)-L-serine (**3**)

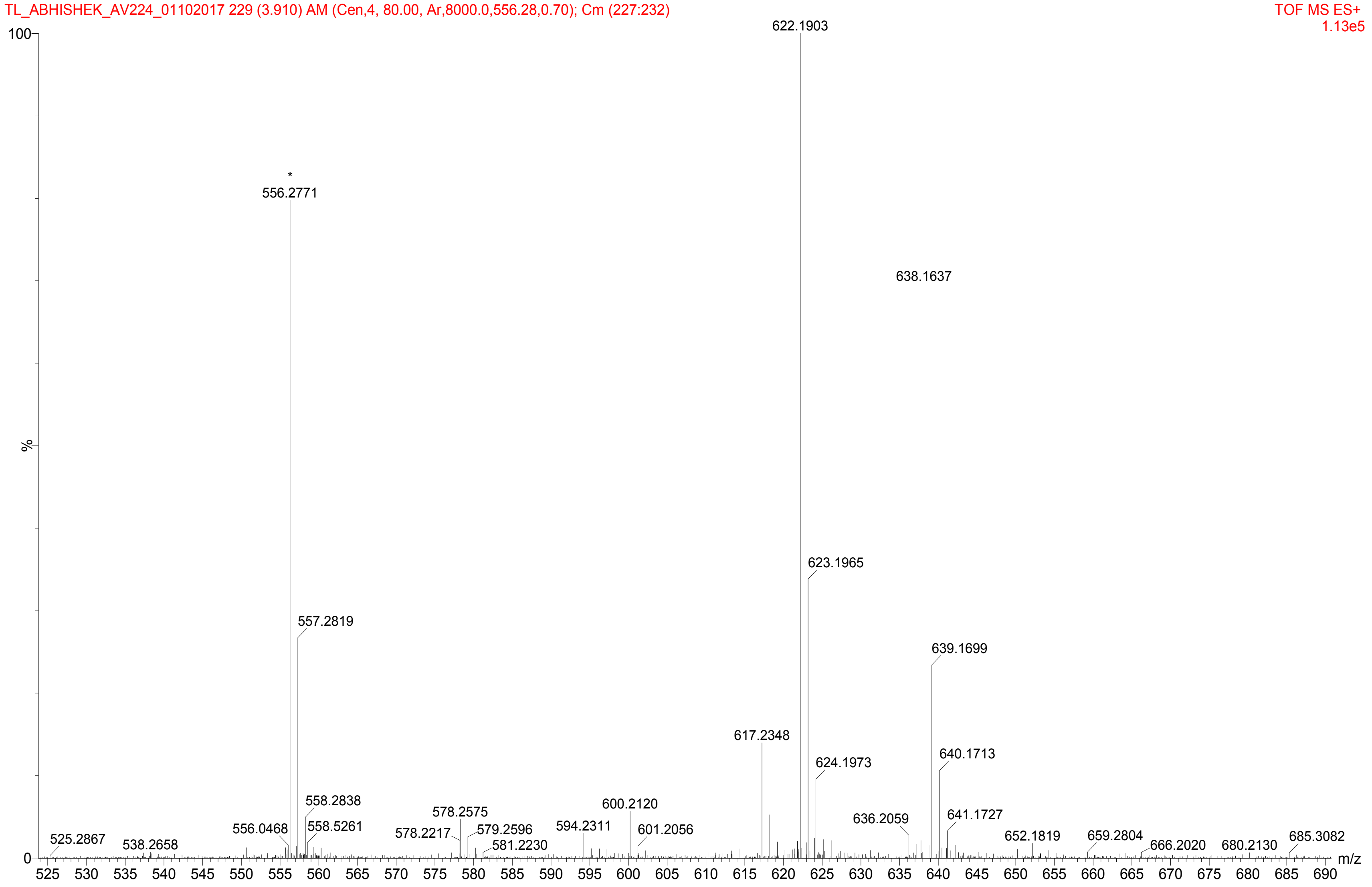

Figure S5. ^{1}H NMR of *N*-[(9H-fluoren-9-yl)methoxycarbonyl]-*O*-((2,3,4-tri-*O*-acetyl)-α-L-rhamnopyranosyl)-L-serine (**3**)

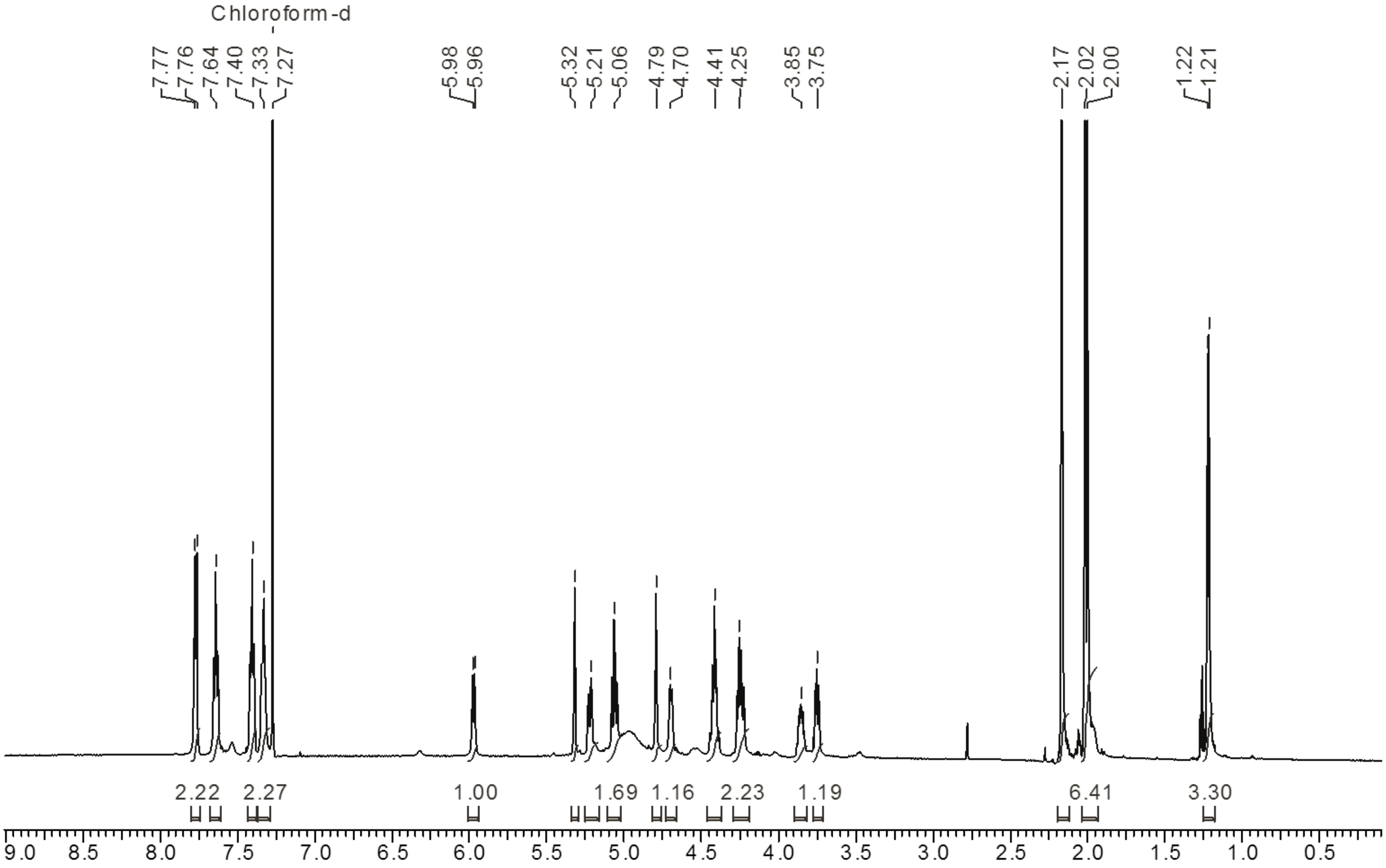

Figure S6. ^{13}C NMR of *N*-[(9H-fluoren-9-yl)methoxycarbonyl]-*O*-((2,3,4-tri-*O*-acetyl)-α-L-rhamnopyranosyl)-L-serine (**3**)

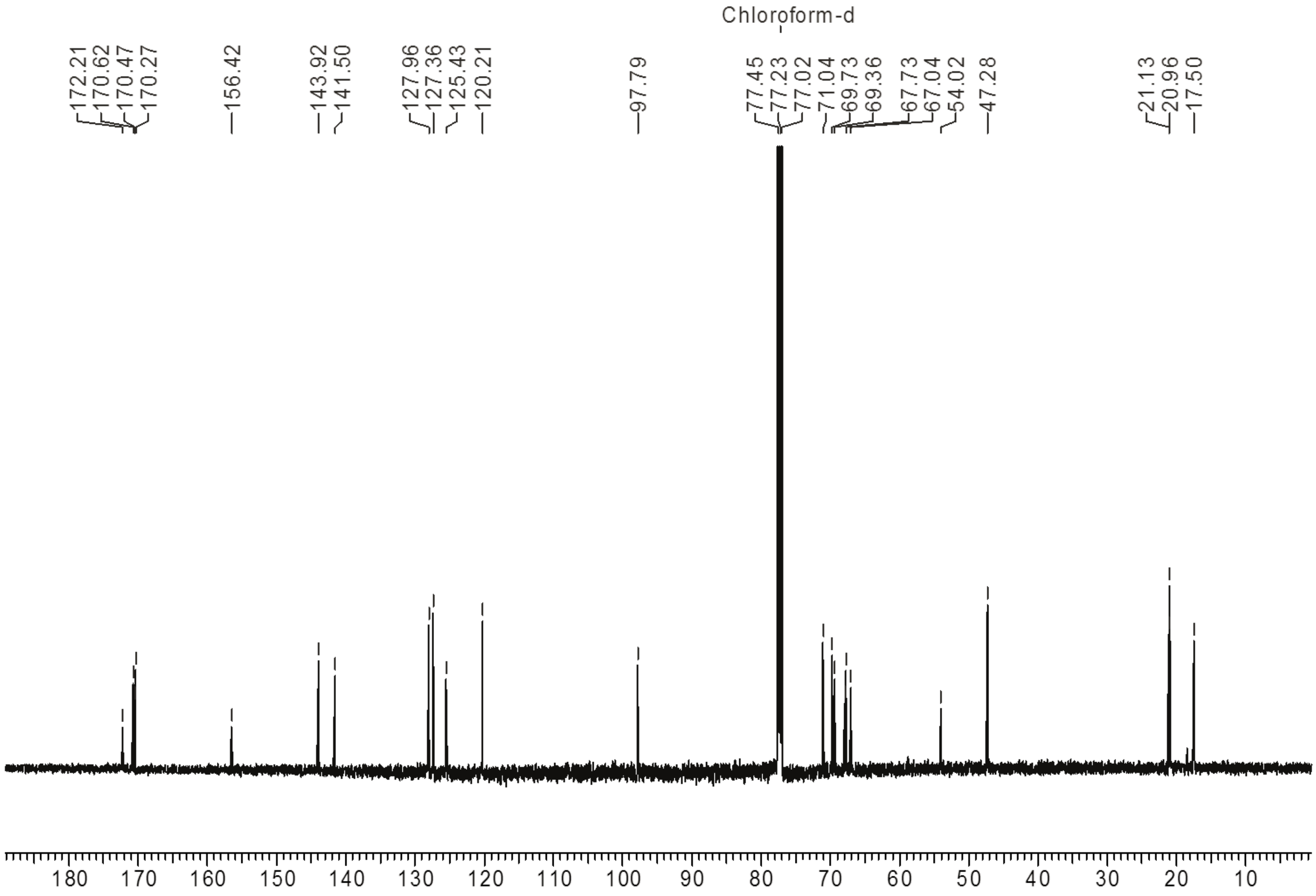

Figure S7. MALDI-TOF data of single molecule construct of Pam_3CSK_4-DBCO-MUC-1 -$(Rha)_3$ sequence (**6**)

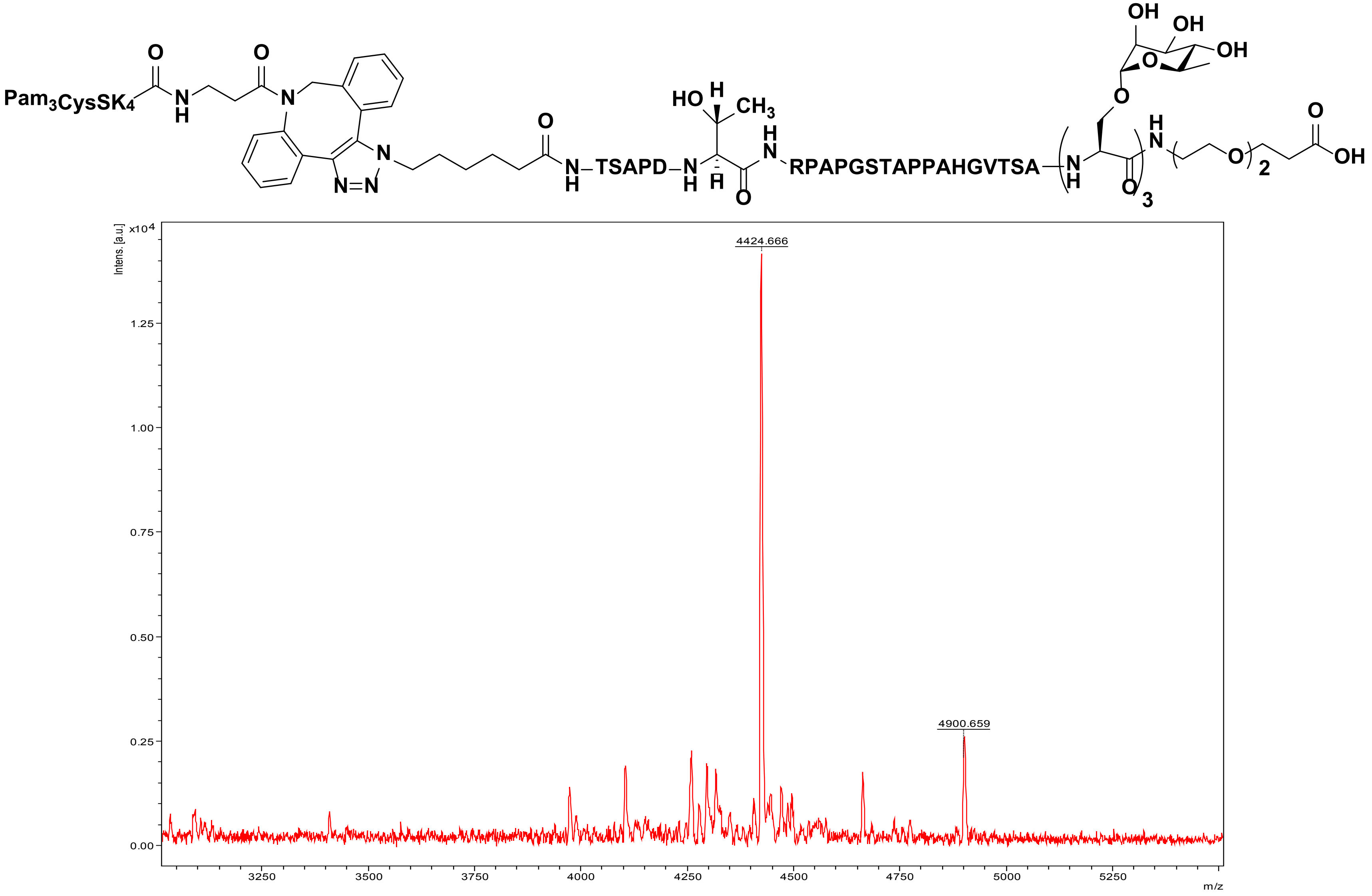

Figure S8. MALDI-TOF data of single molecule construct of Pam_3CSK_4-DBCO-MUC-1 (**8**)

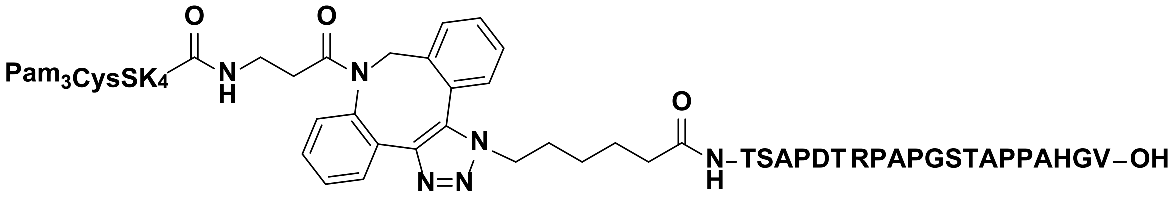

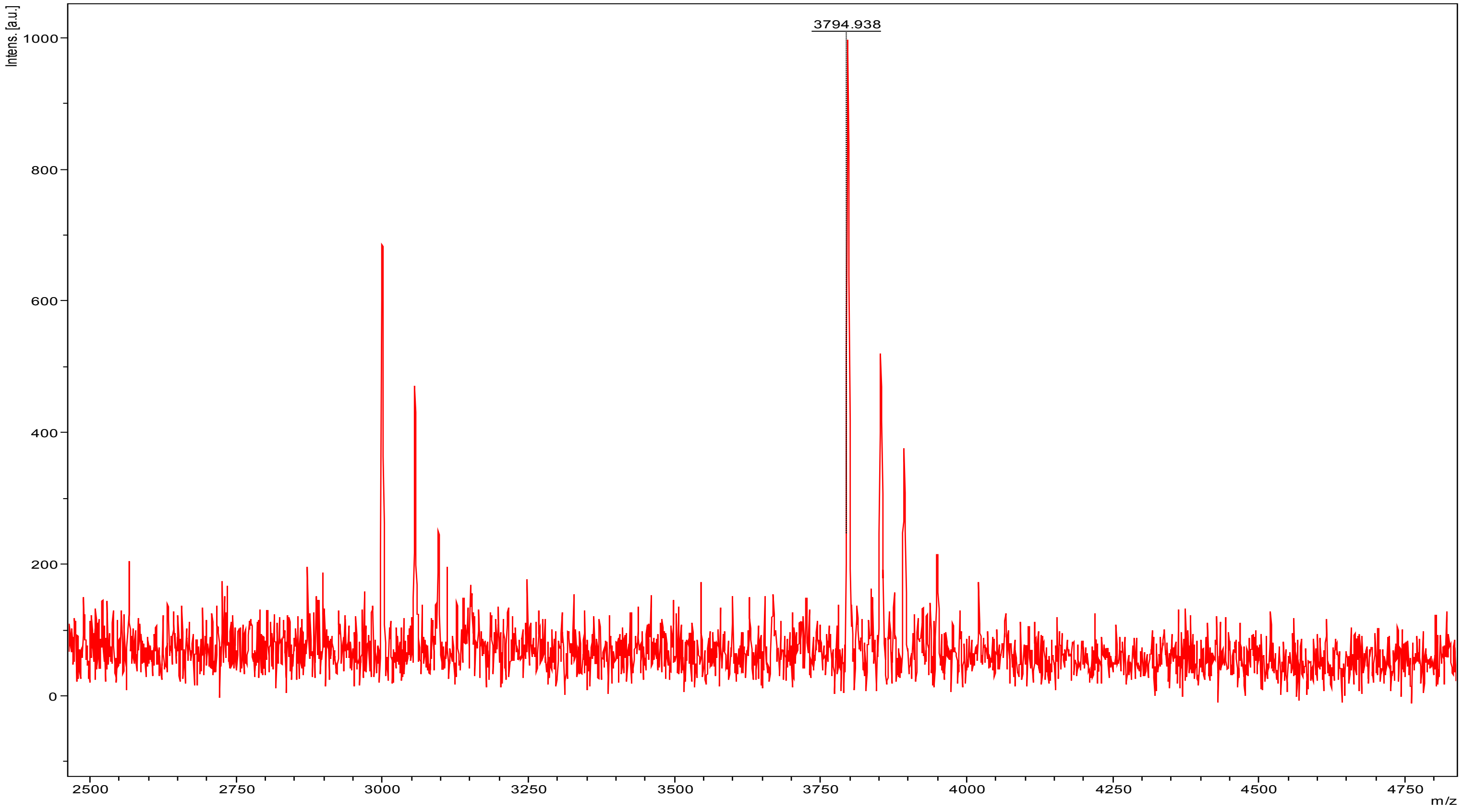

Supplement: Supplementary file 1 [file molecules-25-03137-s001.pdf]
